# Bibliometric Analysis of the Results of Cardio-Oncology Research

**DOI:** 10.1155/2020/5357917

**Published:** 2020-05-13

**Authors:** Kangkang Wei, Jiangquan Liao, Jiangmeng Chang, Xiaoqiong Zhang, Ming Chen, Jinhang Du

**Affiliations:** ^1^Graduate School Beijing University of Chinese Medicine, Beijing, China; ^2^Department of Cardiology of Integrated Chinese and Western Medicine, China-Japan Friendship Hospital, Beijing, China

## Abstract

**Objective:**

To analyze the development of cardio-oncology, summarize the research achievements, and provide proposals for its future research.

**Methods:**

The web of science database was used to search for “cardio-oncology” and “oncocardiology” related articles from the beginning of the database (1970) to April 5, 2019. Excel 2016 and Cytoscape were used to analyze the trend of cardio-oncology research.

**Results:**

A total of 356 articles were obtained. The number of articles has grown rapidly in recent years. Cardiac injury caused by tumor therapy was a research hotspot (*n* = 107). Researchers paid more attention to the prevention and treatment of cardiotoxicity (*n* = 54). Experimental researches were a small part of all studies (*n* = 72), mainly focusing on the study of cancer drugs' cardiac injury, test indicators of cardiotoxicity, and preventive drugs. The United States (*n* = 156.25), Italy (*n* = 48.5), and Canada (*n* = 23.5) published the most articles, making a great contribution to the development of cardio-oncology.

**Conclusions:**

Cardio-oncology has been developing rapidly and receiving a large amount of research efforts in recent years. Most articles on cardio-oncology were published by the authors from the United States (44%) and Italy (17%), while other countries need to pay more attention to cardio-oncology. As an independent discipline, cardio-oncology is certainly in need of significant progress, but it has formed a basic framework, which has obtained many leading theories and meaningful achievements in diagnostic criteria, diagnostic methods, prevention and treatment, mechanism research, and influencing factor. Cardiac injury of tumor drugs has always been a research hotspot in this discipline, and there is still a lot of research space. The research about detection methods of cardiotoxicity and preventive drugs is gradually increasing. Basic research lags behind, and many mechanisms are still unclear.

## 1. Introduction

Cancer and cardiovascular diseases are the main causes of morbidity and mortality in modern society [1]. Cancer mortality is steadily decreasing at a rate of about 1.5% per year [2]. The increase in survival has highlighted the side effects of anticancer therapy, and cardiovascular disease (CVD) is now the leading cause of death among female survivors from breast cancer [3]. Oncohematology treatments increase the risk of cardiovascular events in the mid-to-long term by three times [4–6]. This understanding has contributed to the relationship between cardiologists and oncologists. Their cooperation has formed a relatively new multidisciplinary field called cardiology-oncology. There are two goals of cardio-oncology, one of them is to understand the pathophysiology of cancer treatment associated cardiotoxicity, and the other one is to provide early recognition and treatment of cardiac complications in cancer patients or survivors [7].

Bibliometric analysis is an approach to evaluate the academic impact and characteristics of publications in a specific research field over time [8]. It has played an important role in the past to govern policy making and inspiration in future research, including cardiovascular disease [9], digestive system diseases [10], diabetes [11], coronary heart disease [12], and breast cancer [13]. The rapid development of cardio-oncology to the present has produced many important results, but the research content and development of the subject have not yet been summarized.

In this study, we aimed to provide detailed evaluation of the status of cardio-oncology by using bibliometric analysis, so that more researchers can realize the importance of this field and solve problems that hinder development. At the same time, through the summary of research methods and contents, our study reflected the current situation of the research and provided suggestions for further studies. We hoped to get insights into cardio-oncology which have been gained over the past years by analyzing the publication year, journal, authorship, country and interactions, article type, research content, H-index, citation density, funding source, etc.

## 2. Methods

Web of science database has the advantages of high quality, fast update, and little data error [14]. The web of science database was searched to identify the articles concerning cardio-oncology from the beginning of the database (1970) to April 5, 2019. The search terms were the “topic” (title, abstract, author's keywords, and Key Words Plus) with the following strategy: TS = “cardio-oncology” or “oncocardiology.”

The article of each search result was read thoroughly to ensure that cardio-oncology was the major subject of the research. We excluded literature unrelated to the subject study (*n* = 5), unpublished Meeting Abstract (*n* = 49), errata description (*n* = 1), and literature not available (*n* = 1) and finally obtained 356 articles all related to cardio-oncology. These articles were all from the SCI/SCIE database and have the same weight.

In this study, we analyzed the web of science through Excel, while we showed interactions between countries of articles with the software of Cytoscape. We collected the following information: (1) publication information, including authors, journal, publication year, and author's country; (2) article content, including article type, research content, and funding source; and (3) bibliometric content such as impact factor, citation density, and H-index.

## 3. Results

### 3.1. Language of Publications and Publication Year

**Figure 1:**
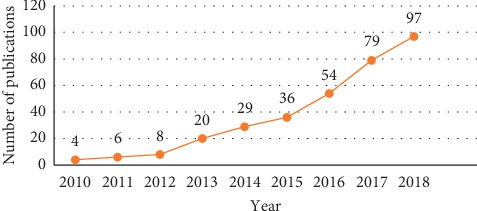
Trends of number of publications.

Among the 356 articles obtained, there are 6 language types, including English (*n* = 343), Spanish (*n* = 4), Portuguese (*n* = 4), Hungarian (*n* = 1), German (*n* = 1), and French (*n* = 3). The earliest articles in the web of science were published in 2010 (*n* = 4), followed by an exponential increase in the number of documents. In 2018, 97 articles were published and this proportion was as high as 27.2%.  Figure 1 shows the number of publications from 2010 to 2019.

### 3.2. Distribution of Citations and H-Index

The number of citations related to cardio-oncology varied from 0 to 240 times, with an average of 10.455 times per article. 275 articles were cited less than 10 times (77.25%), and only 14 articles were cited more than 50 times (3.93%). The article which had the maximum citation (*n* = 240) was “*2016 ESC Position Paper on Cancer Treatments and Cardiovascular Toxicity Developed under the Auspices of the ESC Committee for Practice Guidelines*.” This article discussed nine main categories into which the cardiovascular complications of cancer therapy can be divided. We refer to the number of times that each document is cited every year as the citation density. For the articles which were published in 2018 and beforehand, the citation density ranged from 0 to 80 times, and the average citation density was 4.015 times. There are three articles that have a citation density more than 30 times including two guidelines and one retrospective study and 292 articles (82%) less than 5 times. Similarly, through web of science, we obtained the H-index of the article and analyzed it, excluding 7 articles that could not obtain the H-index. The results showed that the H-index ranged from 0 to 35. The number of articles with H-index of 0 was 136 which occupied the largest proportion (38.2%), and 21 articles with an H-index more than 10 accounted for 5.9% of all publications. The article of the highest H-index (*n* = 35) was “*Cardiovascular Side Effects of Cancer Therapies a Position Statement from the Heart Failure Association of the European Society of Cardiology*” published in 2011.

### 3.3. Distribution of Article Type

**Figure 2:**
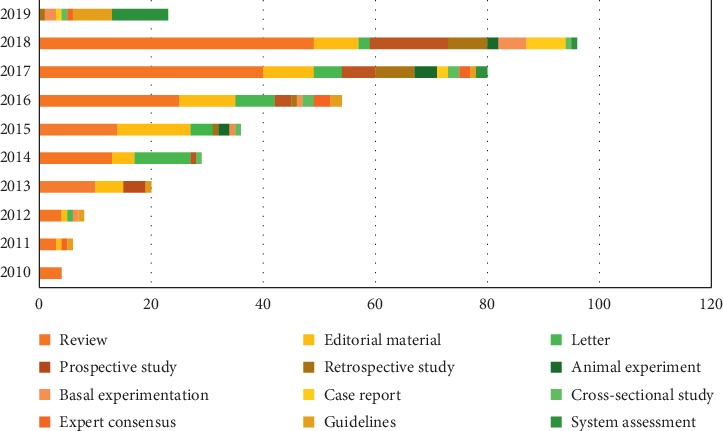
Distribution of article types by year.

The articles on cardio-oncology were classified by article type, including review, editorial material, letter, prospective study, retrospective study, animal experiment, basal experimentation, case report, cross-sectional study, expert consensus, guidelines, and system assessment. There are 173 review articles and 72 articles about experimental study (prospective study = 29, retrospective study = 17, animal experiment = 10, basal experimentation = 9, and cross-sectional study = 7).  Figure 2 shows the publication of different literature types from 2010 to 2019.

### 3.4. Distribution of Study Content

**Figure 3:**
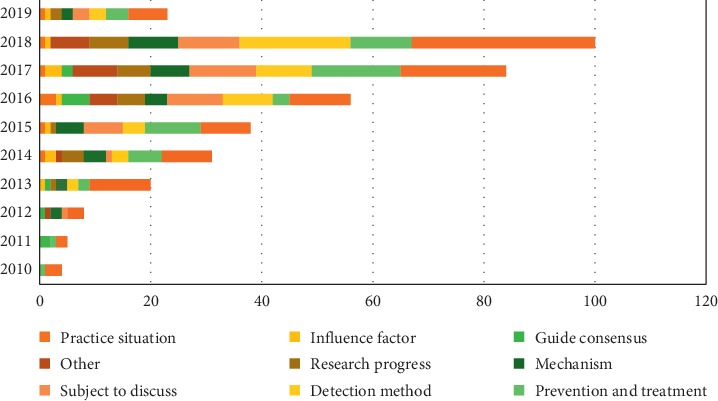
Distribution of study contents by year.

The articles on cardio-oncology were classified according to the study content, including cardiotoxicity, prevention and treatment, detection method, subject to discuss, mechanism, research progress, consent consensus, influence factor, and practice situation. Among them, cardiotoxicity had the largest number (*n* = 107), followed by prophylaxis and treatment (*n* = 54) and detection method (*n* = 51).  Figure 3 shows the publication of different study contents from 2010 to 2019.

### 3.5. Distribution of Output in Journals

Literatures of cardiac-oncology were published in 148 different journals. All publications were mainly published in cardiovascular journals, and cancer journals were not prominent. The journals with more than 5 articles were listed in Table 1.

### 3.6. Contribution Distribution of Countries

The authors of the cardio-oncology articles were from 33 countries. In all articles, 309 articles were written by authors from single country, 31 articles by authors from 2 countries, and 15 articles by authors from three or more countries. In order to show the number of articles published in each country more accurately, we used the integration method. Each article scored 2 points, including 1 point for the first author and 1 point for the correspondent author. For example, the first author of an article is Italy, and corresponding author is the United States; then Italy and the United States each scored 1 point and finally divided by 2. In this way, the number of articles published in each country was obtained. After excluding an article by a group author and an article without a country, the total score was 354; the highest score was gained by USA (*n* = 156.25), followed by Italy (*n* = 59.5), Canada (*n* = 23.5), Germany (*n* = 16), and Japan (*n* = 14). The top 10 countries were ranked (Table 2).

### 3.7. Publication Distribution of Institutes and Author

Cardiologia Ospedale San Vincenzo in Italy was the most prolific research institute in cardio-oncology (*n* = 13), followed by University Hospital Essen (*n* = 6) in Germany, The University of Texas (*n* = 5) in the United States, Aix-Marseille University (*n* = 4) in France, Mayo Clinic (*n* = 4) in the United States, and Shizuoka Cancer Center (*n* = 4) in Japan. In the articles on cardio-oncology, a total of 67 authors published more than one article as the first author or correspondent author. There were 8 authors who published more than 4 articles, and, among them, Salvatore Patanè from Italy was the most productive author (*n* = 13).  Table 3 shows the authors with more than 4 articles.

### 3.8. Distribution of Funding Agencies

In the 356 articles, 229 articles (64.3%) recorded not receiving any funding. Only 127 articles (35.7%) were funded by 229 funding agencies. The United States provided the most funding and had the most funding agencies.  Table 4 shows the funding agencies that provided funding more than twice.

### 3.9. Distribution of Interactions between Countries

**Figure 4:**
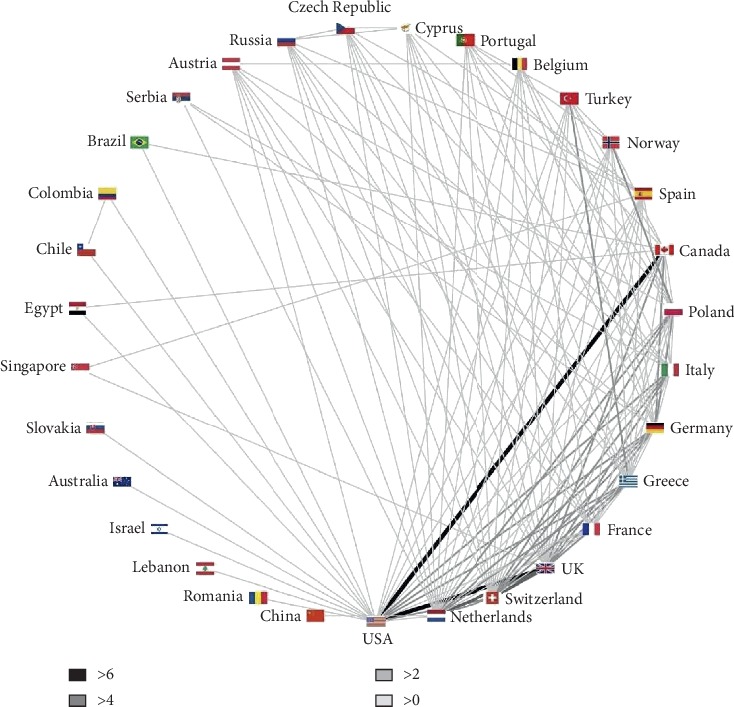
Interactions between countries of articles on cardio-oncology.

According to analysis of national cooperation, the cooperation between the USA and Canada was the most frequent (*n* = 8), followed by USA-UK (*n* = 7), UK-Netherlands (*n* = 6), and UK-Switzerland (*n* = 5).  Figure 4 shows the interactions between countries of articles on cardio-oncology.

## 4. Discussion

Cardio-oncology has become an influential, rapidly developing, and important translational research area in modern medicine, which is getting more and more attention [15, 16]. Cardiotoxicity can be caused by cytotoxic chemotherapy, radiotherapy, molecular targeted therapies, and immune-modulating agents. At present, the cardio-oncology researches mainly focus on pathophysiological mechanisms in the development of heart failure, risk factors, and early signs of cardiotoxicity detectable, as well as cardioprotective treatments [17].

In this study, we reviewed and analyzed the articles on cardio-oncology. Through bibliometric analysis, we can understand the development and research status of cardio-oncology and provide reference for future development and research direction. The first article on cardio-oncology in the web of science was published in 2010. Although cardio-oncology is a new subject with few publications, we can see that the number of articles has grown exponentially. Therefore, more and more attention has been paid to this subject and it will remain a hot research topic for the foreseeable future.

Most of the articles on cardio-oncology were cited less than 10 times (*n* = 275), and the average citation density was 4.015 times. There were 21 articles with an H-index that is greater than 10, while the H-index in 136 articles (38.20%) is 0. We speculate that the number of studies in this discipline is relatively small or there are more new researches, resulting in low level in the number of citations. 12 of 21 articles with H-index >10 were cited more than 50 times, and, among them, three articles were consensus guidelines, which summarized the current research results of cardio-oncology in detail and introduced the cardiovascular diseases caused by radiotherapy, chemotherapy, and targeted treatment. These three articles also provided suggestions for the prevention and treatment of cardiovascular diseases. In addition, there were also two articles about the drugs with cardiotoxicity, two articles reviewing the prevention and treatment of cardiotoxicity, two articles discussing the mechanism of cardiotoxicity, two articles describing the experiment of cardiotoxicity leading to the change of cardiac biomarkers, and one article about the treatment of tumor combined with atrial fibrillation. In conclusion, researchers were more concerned about the cardiotoxicity of cancer drugs and how to prevent cardiac damage.

72 articles (20.22%) were experimental research; among them, 29 articles (40.28%) were prospective studies. The percentage was small, but, in recent years, the experimental research was growing rapidly, and the proportion in 2018 reached 30.21%. It can be seen that although experimental researches were relatively few, they have gained attention by scholars in recent years. These experimental studies can be divided into 6 categories according to the content. Firstly, 25 articles studied the cardiovascular injury caused by tumor treatment via different experiment methods. Secondly, 17 articles explored the indicators of early detection and monitoring of cardiotoxicity, including biomarkers and imaging examination. Thirdly, 9 articles discussed the drugs to prevent cardiotoxicity. In addition, 8 articles studied the mechanism of cardiotoxicity. Moreover, 6 articles investigated the development of cardio-oncology in different regions. Furthermore, 5 articles reported the interaction between tumor and cardiovascular disease. Unfortunately, there was no research result about radiotherapy. Another feature of all publication was that the proportion of review article is 48.31%. These review articles described cardiovascular damage and recent research progress in chemoradiotherapy. Cardio-oncology serves as a bridge between heart disease and oncology [18]. Many experiments in heart disease or in oncology have been completed before cardio-oncology established. The emergence of cardio-oncology provides better integration of these results.

In terms of research content, these studies included prevention and treatment, detection method, mechanism, and influence factor, which constitute the skeleton of the discipline. 30.06% of articles were about cardiotoxicity in tumor treatment, reflecting the research direction of the discipline. We could find that the previous research focused on three aspects. First of all, cancer drugs that are prone to cardiac damage were the core of the research. In the early stage, anthracyclines were mainly studied. Recently, the research about monoclonal antibodies and targeted drugs is gradually increasing. Furthermore, main research drug of cytotoxic drugs was cyclophosphamide. Other sorts of drugs, such as hormones, were less studied. Secondly, at present, monitoring the indicator of early cardiotoxicity is a hotspot. Early detection of cardiac impairment is an important step in adjusting tumor medication and giving prevention and treatment of angiocardiopathy. It not only confirmed the role of traditional indicators such as LVEF and cardiac troponin, but also found indicators such as global longitudinal strain (GLS) and myeloperoxidase (MPO) that respond to early cardiac damage. Finally, drugs to prevent cardiotoxicity will be the focus of future research. Currently, preventive effects of drugs have been proved, and they are generated by Angiotensin-Converting Enzyme Inhibitor (ACEI), Angiotensin Receptor Blocker (ARB), and *β*-blocker such as perindopril, valsartan, metoprolol, and bisoprolol. These medicines are expected to be widely used in future clinical practice. Besides, 12.92% of the articles discussed the importance of the subject, the direction of development, and how to develop the subject, affirming the importance of cardio-oncology. It is worth noting that, in others, we can see some experts who have studied the relationship between cardiovascular disease and tumor disease. The research content of cardio-oncology is not limited to the toxicity of radiotherapy and chemotherapy to the heart. The study of disease similarities in two disciplines contributes to the development of cardio-oncology.

The articles on cardio-oncology were published in 148 journals, including cardiovascular disease journals, oncology journals, and comprehensive magazine. 62.16% of journals published only one literature, and 16 journals published more than five cardio-oncology articles.

In the articles on cardio-oncology, the authors from United States contributed 44%, far exceeding other countries, followed by Italy with 17%, Canada with 7%, and Germany with 5%. The United States leads the world in the field of cardio-oncology research, which is consistent with its leading position in many other fields. However, it is regrettable that there are still many countries with insufficient knowledge of this subject and few research results.

A total of 31 countries had cooperated with other countries and formed 138 kinds of cooperation between two countries. There are 70 types of cooperation that happened once, accounting for 51.06%, and 42 kinds of cooperation (30.66%) happened twice. In all countries, the United States had more cooperation than anyone else, and the frequency of cooperation between USA and Canada is the highest which is 8 times in total. European countries such as Italy, Britain, Canada, and The Netherlands had also cooperated many times, while cooperation among nations in Asia, Africa, and South America rarely appeared. International cooperation articles are often more significant than other articles [19]. Many major studies require the joint efforts of many countries. The development of cardio-oncology needs to further strengthen cooperation among countries. We hope that developed countries can fully exert their enthusiasm and drive the surrounding areas. These countries will provide impetus for the further development of cardio-oncology.

Salvatore Patanè from Italy had the largest number of published articles, and his organization, Cardiologia Ospedale San Vincenzo, was the institution with the highest amount of literature. Corresponding to the national contribution, the authors who published more than 4 articles are American or Italian. The NIH, NHLBI, and NCI in the United States funded 49 studies; hence the United States had the maximum number of funding this research. Cardio-oncology has not been funded by large pharmaceutical companies, which may be related to its research content. Pharmaceutical companies may not be willing to provide additional expenditures for the study of drug side effects. Therefore, the development of cardio-oncology needs more help from government.

As the USA provided the most funding, this reflects the strong interest of US authors and funding agencies in the discipline, and, in the future, this may continue to contribute to the development of cardio-oncology and have a positive international impact.

Cardio-oncology has entered a period of rapid development. Some countries have launched related services, but that cannot meet the needs. As early as 1975, there were reports of cardiotoxicity of Daunomycin and Adriamycin [20, 21]. However, it was not until 2000 that Anderson established the world's first association of cardio-oncology; the International Cardio-Oncology Society (ICOS) was founded in 2009. After the establishment of ICOS, cardio-oncology began to develop rapidly. In 2010, web of science published the first article on cardio-oncology. Investigating the development of cardio-oncology in various countries, the survey of the Tuscany region in Italy at the end of 2011 revealed that only the minority offered to outpatients a cardio-oncology service (20%) or possessed a trained cardiology team (28%) [22]. In 2015, an international survey of cancer centers showed that cardio-oncology clinics were run in 39 out of 113 (35%) centers [23]. The results of a survey of 303 oncologists in France in 2016 showed that three hundred and one oncologists (99%) had prescribed potentially cardiotoxic cancer therapies, but 64% of oncologists have unclear understanding of the cardiotoxicity of cancer treatment [24]. The practice of cardio-oncology still needs to be improved. In terms of national contribution, research on cardio-oncology was mainly concentrated in countries in Europe and in the United States. Countries in Asia, Africa, South America, and other geographic areas had too little research in this field. These countries need to enhance the understanding of chemoradiotherapy for heart damage and translate the research results into timely clinical benefit. There are 7998 articles on esophageal cancer in 2012–2016 [25]. After searching the web of science and PubMed from its establishment to the present, we found that there were only 1173 articles about cardio-oncology, in which Europe and America were the dominant countries. The research of cardio-oncology was still obviously inadequate, compared with the research of traditional cancer field, and the research results were mainly concentrated in European and American countries, while the research in other countries was seldom. We compared 302 articles on oncology published by The National Cancer Database (NCDB). These articles were cited 9858 times totally, and the average citation frequency of an article was 32.6 [26], while the average citation frequency of cardio-oncology was 10.46. Cardio-oncology deserves greater attention.

Reviewing the recent research results, the cardiotoxicity of conventional drugs such as Anthracyclines, cyclophosphamide, and Trastuzumab has been clearly recognized. In recent years, the rapid development of emerging therapies, such as immunotherapy and targeted therapy, has played an increasingly important role in anticancer therapy. The cardiotoxicity brought by these therapies has also aroused attention, and new research results in these fields are being published [27, 28]. The guidelines of European Society of Cardiology introduced detailed research results of nine cardiovascular damage studies in 2016. American Society of Clinical Oncology summarized previous research findings and discussed detection methods, prevention and treatment, assessment, and management methods in 2017. However, the specific mechanisms by which radiation or drugs cause cardiovascular disease have not been elucidated. Cardiovascular assessment and monitoring still cannot be resolved. In addition, there is still insufficient treatment of cardiovascular side effects and evidence of the role of cardiovascular factors in cardio-oncology is still lacking [15, 29, 30]. Through our research, we could find that cardio-oncology has a comprehensive system in terms of cardiotoxic drugs, monitoring cardiotoxic indicators, and preventive drugs, which plays a certain role in guiding clinical practice. Researchers have reported on the cardiovascular toxicity of many types of drugs such as monoclonal antibodies, targeted drugs, endocrine drugs, and antiangiogenic drugs, but the number of studies of each type of drug was relatively small. Furthermore, the trials focused too much on several drugs that had been reported and lacked exploration of other drugs that might have cardiotoxicity. At present, cardio-oncology is dominated by clinical research, mainly focusing on the cardiovascular damage of drugs, detection methods of cardiac injury, and drugs for preventing damage. Basic research on cardio-oncology is inadequate and many mechanisms are unclear. Interdisciplinary study may create some difficulties. The leading role of clinical research results in that researches of cardio-oncology focus on diagnosis and treatment. In fact, basic research is the inexhaustible driving force for its long-term development.

With regard to cardiac dysfunction caused by tumor treatment, research on heart failure is relatively perfect. There is a basic consensus in terms of toxic drugs, detection methods, diagnosis methods, and prevention and treatment schemes. However, focusing too much on heart failure led to the lack of study, which investigated other kinds of cardiovascular diseases. Research studies about arrhythmia or myocardial damage caused by tumor treatment were far less rich than the study of heart failure. Indeed, heart failure is the main manifestation of cardiovascular injury and hotspot of research, but we should strengthen our understanding of cardiovascular disease other than heart failure, especially to make up for the deficiency of research on detection methods and preventive drugs.

We still face many challenges in the future. As new chemotherapeutic drugs continue to be produced, reasonable cardiotoxicity assessments and prevention programs for various chemotherapy regimens require more funding. We need to further standardize cardiotoxicity detection methods. We also need to clarify the time when to start the prevention of cardiotoxicity to have the greatest benefits and to fill gaps in the study of multiple chronic conditions. We hope that researchers will recognize that multidisciplinary cooperation is a necessary condition for the development of cardio-oncology [15, 30, 31].

Our research had certain limitations. The content we searched for was only the web of science. It did not include other databases such as PubMed, paper publications, and video materials. Our search terms were “cardio-oncology” and “oncocardiology,” so it was unavoidable that some articles might be missed. We had not included these relevant contents into the research, and there was a certain deviation. There may be many factors that affect the citation numbers of an article, and the citations cannot fully reflect the academic impact of an article. The United States had the largest number of articles, but the article that had been cited most highly was from Europe, and the author who published the most articles was Italian. Although we analyzed cardio-oncology as comprehensively as possible, it was still difficult to avoid bias. The density of citations is greatly influenced by the development of the discipline. The earlier the article is published, the more the times it is cited, and the new research results published in recent years are less frequently cited. Although the H-index compensates for the deficiency to a certain extent, it is still difficult to perfectly capture the impact of new research results. Despite this, we have analyzed the development and research contents of cardio-oncology as comprehensively as possible, with a view of summarizing this subject and providing a certain directive role in the period of rapid development of cardio-oncology.

## 5. Conclusion

In this study, we used bibliometric methods to analyze the articles on cardio-oncology and summarized the research progress of this discipline. The establishment and development of a discipline require core research (cardiotoxicity), diagnostic criteria, diagnostic methods, prevention and treatment, mechanism research, influencing factors, and disciplinary development planning. The study of cardio-oncology has these conditions but needs improvement. Researchers mainly focus on cardiovascular damage of tumor drugs. It would still be a hotspot in the future, because there are still many drug toxicities that have not been investigated. With the continuous report of cardiotoxicity of tumor drugs, how to monitor cardiotoxicity and prevent heart damage will be a new hotspot. Previous studies focused on heart failure, while there was insufficient research on other cardiovascular diseases. Unfortunately, many problems were found in the clinic rather than basic research. Hence, basic research failed to provide beneficial support for clinical research. It is because many problems were found in the clinic rather than basic research that basic research failed to provide beneficial support for clinical research. We hope that as the research results continue to be enriched, a system would be established to guide the use of cardiotoxic drugs, cardiotoxic monitoring, and the timing of initiation of cardiovascular disease prevention.

## Figures and Tables

**Table 1:** Journals published more than five times (sorted by number of articles).

Journal	Impact factor (2018)	No. of article(s)
*International Journal of Cardiology*	3.472	30
*Journal of the American College of Cardiology*	18.639	21
*Future Oncology*	2.279	13
*JACC-Cardiovascular Imaging*	10.975	11
*Current Cardiology Reports*	2.395	9
*Current Oncology Reports*	3.949	9
*Circulation*	23.054	8
*Heart Failure Clinics*	1.895	8
*Internal and Emergency Medicine*	2.335	8
*Journal of Cardiac Failure*	3.875	8
*Journal of Thoracic Disease*	2.027	8
*Journal of Nuclear Cardiology*	4.112	7
*European Heart Journal*	23.239	6
*European Journal of Heart Failure*	13.965	6
*Journal of the American Heart Association*	4.660	6
*Mayo Clinic Proceedings*	7.091	6

**Table 2:** Top ten productive countries publishing cardio-oncology articles.

Country	Number of articles	Percentage
USA	156.25	44.1%
Italy	59.5	16.8%
Canada	23.5	6.6%
Germany	16	4.5%
Japan	14	4.0%
UK	11	3.1%
France	10	2.8%
Spain	7.75	2.2%
Greece	7	2.0%
The Netherlands	7	2.0%
Others	42	11.9%

**Table 3:** Authors with more than 4 articles.

Author	No. of articles	Affiliation	Country
Salvatore Patanè	13	Cardiologia Ospedale San Vincenzo	Italy
Daniel J. Lenihan	7	Vanderbilt University Medical Center	USA
Bonnie Ky	6	The University of Pennsylvania	USA
Ana Barac	6	Georgetown University	USA
Michael G. Fradley	5	University of South Florida	USA
Joerg Herrmann	5	Mayo Clinic	USA
Giorgio Minotti	5	University Campus Bio-Medico, Rome	Italy
Cezar Iliescu	5	University of Texas	USA

**Table 4:** Funding agencies with more than two times of funding.

Funding agencies	Number
National Institutes of Health	37
National Heart, Lung, and Blood Institute (NHLBI)	10
National Cancer Institute (NCI)	9
Bristol-Myers Squibb	5
Associazione Italiana per la Ricerca sul Cancro (AIRC)	4
American Heart Association	4
Canadian Institutes of Health Research	3
Sanofi Aventis	3

## Data Availability

Our data are from the analysis of articles and can be provided when necessary.
